# Altered Regional Homogeneity in Patients With Corneal Ulcer: A Resting-State Functional MRI Study

**DOI:** 10.3389/fnins.2019.00743

**Published:** 2019-07-23

**Authors:** Man-Wei Xu, Hui-Min Liu, Gang Tan, Ting Su, Chu-Qi Xiang, Wei Wu, Biao Li, Qi Lin, Xiao-Wei Xu, You-Lan Min, Wen-feng Liu, Gui-Ping Gao, Yi Shao

**Affiliations:** ^1^Department of Ophthalmology, The First Affiliated Hospital of Nanchang University, Jiangxi Province Ocular Disease Clinical Research Center, Nanchang, China; ^2^Department of Ophthalmology, University of South China, Hengyang, China; ^3^Eye Institute of Xiamen University, Fujian Provincial Key Laboratory of Ophthalmology and Visual Science, Xiamen, China

**Keywords:** corneal ulcer, regional homogeneity, functional MRI, resting state, brain activity

## Abstract

**Objective:**

To investigate the potential regional homogeneity (ReHo) brain activity changes in patients with corneal ulcer (CU) and their possible relationship with clinical symptoms.

**Materials and Methods:**

Forty patients with CU (26 men and 14 women), and 40 healthy controls (HCs) (26 men and 14 women) closely matched in age, sex, and weight underwent resting-state functional MRI scans, respectively. The ReHo method was applied to evaluate synchronous neural activity changes. Receiver operating characteristic curve (ROC curve) was used to show high test-retest stability and high degree of sensitivity and specificity. We utilized the correlation analysis to calculate the relationship between the average ReHo signal values in different brain areas and the clinical symptoms in CU patients.

**Results:**

Compared with the HCs, CU patients had significantly increased ReHo values in right cerebellum posterior lobe, left cerebellum posterior lobe, left inferior temporal gyrus, right lingual gyrus, left middle frontal gyrus, left angular gyrus, left cingulate gyrus, right angular gyrus and bilateral superior frontal gyrus, and decreased ReHo values in right anterior cingulate and left precentral gyrus. ROC curve analysis of each brain regions showed the accuracy of AUC was perfect except the right cerebellum posterior lobe. Nevertheless, there was no clear evidence of prominent relevance between the average ReHo values in brain areas and the clinical symptoms.

**Conclusion:**

Corneal ulcer caused dysfunctional adaption in different brain areas, which including relatively increased values and decreased values. This finding may help us take a further step in exploring the underlying pathologic mechanisms of CU.

## Introduction

Corneal ulcer (CU), a common ophthalmological disease, is a kind of inflammatory reaction of cornea. In worldwide, corneal disease is the fifth leading cause of blindness, following cataract, refractive error, glaucoma, and age related macular degeneration (Flaxman et al., 2017). According to etiology, CU can be divided into infectious CU and non-infectious CU and in China, the infectious factors of virus, fungus, bacteria, and acanthamoeba, plays the leading role in resulting in CU (Song et al., 2011). The morbidity of infectious CU is 0.148% in middle-China (Langkide et al., 2002).

Common symptoms of CU are massive pain, blepharospasm, photophobia, and tearing (Herz et al., 2008). If diagnose delayed, CU can result in severe complications including corneal perforation, endophthalmitis, and iris atrophy (Herz et al., 2008; Rana et al., 2015; Zapp et al., 2018). The tentative diagnosis of CU can often be made by slit lamp, fluorescein staining and *in vivo* confocal microscopy (IVCM) (Herz et al., 2008). A previous research already showed that corneal disease had influence on brain area activities (Arrigo et al., 2018). Nevertheless, the inspection methods mentioned above only focused on the effects of the ocular surface on brain activities and ignored the rest of the visual system containing the eye and the connecting pathways through the visual cortex and other regions of brain.

Synchronous neuronal activity occurs in the normal human brain (Sturm and König, 2001). Synchronous neuronal activity plays a critical role in learning and memory (Jutras and Buffalo, 2010). In addition, reliable propagation of synchronous neuronal activity was shown to be crucial for neuronal information processing (Bayati et al., 2015). Several previous electroencephalographic and functional magnetic resonance imaging (fMRI) studies indicated that synchronous neuronal activity might have a critical role in neurophysiological activity (Ward, 2003; Spencer et al., 2004; Li et al., 2015). The regional homogeneity (ReHo) is widely used to investigate the local synchronization of spontaneous fMRI signals. It has been successfully applied to previous researches about evaluating the brain activities in patients with ocular diseases (Cui et al., 2014; Song et al., 2014; Shao et al., 2015; Huang et al., 2016a, b, 2017a, b). Our study was to determine whether the CU patients were associated with abnormal synchronous neuronal activity related to eye pain perception and visual loss.

## Materials and Methods

### Subjects

The Ophthalmology Department of the First Affiliated Hospital of Nanchang University enlisted 40 CU patients (26 men and 14 women). The inclusion criteria of the study in CU patients were (1) the duration of CU was at least 14 days; and (2) there were no other ocular diseases in any of bilateral eyes (cataracts, glaucoma, retinal degeneration, amblyopia, strabismus, optic neuritis, etc.).

Corneal ulcer with the following conditions were excluded from the study: (1) impending perforation, corneal perforation and blindness; (2) ocular trauma; (3) systemic diseases including hypertension and heart disease; (4) psychiatric diseases; and (5) other disorders that would affect the ReHo measurement.

Totally 40 healthy controls (HCs, 26 men and 14 women) participated in the study and had no statistic differences in age, sex and weight level with patients of CU group. All the HCs met the following criteria: (1) no neurological or psychiatric disorders (Parkinson’s disease, manic depression, depressive disorder and so on); and (2) be qualified to undergo MRI scanning (e.g., no metallic false teeth, cardiac pacemaker or other implanted metal devices, and so on).

The experiment was approved by the First Affiliated Hospital of Nanchang ethics committee. All the methods applied in the research followed the Declaration of Helsinki and complied with the medical ethics. For each subject, the research pact and process were fully explained, and written agreement was acquired.

### MRI Parameters

Magnetic resonance imaging scanning was carried out on a 3-T MR scanner (Trio, Siemens, Munich, Germany). High-resolution *T*_1_-weighted images were obtained with a three-dimensional spoiled gradient-recalled sequence, using the following parameters: repetition time = 1900 ms, echo time = 2.26 ms, thickness = 1.0 mm, gap = 0.5 mm, acquisition matrix = 256 × 256, field of view = 250 mm × 250 mm, and flip angle = 9°. Functional images with the parameters (repetition time = 2,000 ms, echo time = 30 ms, thickness = 4.0 mm, gap = 1.2 mm, acquisition matrix = 64 × 64, flip angle = 90°, field of view = 220 mm × 220 mm, 29 axial) were corrected.

### fMRI Data Analysis

First, all the images were checked by MRIcro^1^ to delete deficient ones. Then we preprocessed the rest of the images by SPM8^2^ and DPARSF^3^ software. Because there was time for the participants get used to the starting noise, we discarded the first 10 volumes of each subject. Then, applying slice timing, head motion correction (within 1.5 mm or 1.5° in any of six parameters) and spatial normalization to the data. The next step is smoothening the data with a Gaussian kernel of 6 mm^3^ × 6 mm^3^ × 6 mm^3^ full width at half-maximum (FWHM). Finally, with the echo-planar imaging template, the fMRI images were spatially standardized to the Montreal Neurological Institute (MNI) space and resampled at a resolution ratio of 3 mm × 3 mm × 3 mm. After all the steps above, for reducing the effects of low-frequency drift, physiological high-frequency respiratory and cardiac noise, the fMRI data were detrended and bandpass-filtered (0.01–0.08 Hz) finally.

Based on Kendall’s coefficient of concordance (KCC), the data of ReHo values was dealt with REST^4^ software to analyze the correlation. The KCC can give voxels through calculating the KCC of time series of the voxel and nearest-neighbor ones (26 voxels): W=∑(R_i_)^2^−n(R)^2^2K^2^(n^3^−n)/12, where W represents the KCC among given voxels, ranging from 0 to 1; R_*i*_ represents the sum rank of the time point; R=(n + 1)K/2 is the mean of the R_*i*_*sK* is the number of time series within a measured cluster (in the study, *K* = 27, plus one voxel located in the cubic center); n is the number of ranks. There, all the ReHo data were generated.

### Statistical Analysis

The fMRI data were examined by two-sample *t*-test with the SPM 8 software (two-tailed, voxel-level: *P* < 0.01, GRF correction, cluster-level: *P* < 0.05) and it helps to compare two group differences in the zReHo maps using the GRF method which was used to correct for multiple comparisons and regressed covariates of age and sex.

### Brain–Behavior Correlation Analysis

According to the ReHo calculation result, some different brain regions showed different signals between CU groups and HCs. For each region, the mean ReHo values was calculated by averaging over all voxels. The relationship between the mean ReHo value and their clinical features was calculated using the correlation analysis (the threshold was set at 0.001, *P* < 0.001 was considered statistically significant).

### Clinical Data Analysis

All the accumulated clinical data were collected, including the course of the disease and the best-corrected visual acuity. The demographic and clinical variables between CU and HC groups were compared using SPSS20.0 software (SPSS, Chicago, IL, United States) with independent sample *t*-test, and *p* < 0.05 was considered to have statistical significance.

To show high test-retest stability and high degree of sensitivity and specificity, we applied the receiver operating characteristic (ROC) curve method.

## Results

### Demographics and Visual Measurements

There were no obvious differences in weight (*P* = 0.892) and age (*P* = 0.824) between two groups. Details are shown in Table 1.

**TABLE 1:** Conditions of participants included in the study.

**Condition**	**CUs**	**HCs**	***t***	*P*-value^*^
Male/female	26/14	26/14	N/A	>0.99
Age (years)	51.25 ± 5.46	51.98 ± 5.18	0.251	0.824
Weight (kg)	63.12 ± 7.35	63.89 ± 6.73	0.181	0.892
Handedness	40R	40R	N/A	>0.99
Duration of CU (days)	25.75 ± 5.65	N/A	N/A	N/A

^*^P < 0.05 Independent *t*-tests comparing two groups. HCs, healthy controls; N/A, not applicable; CU, corneal ulcer.

### ReHo Differences

**FIGURE 1:**
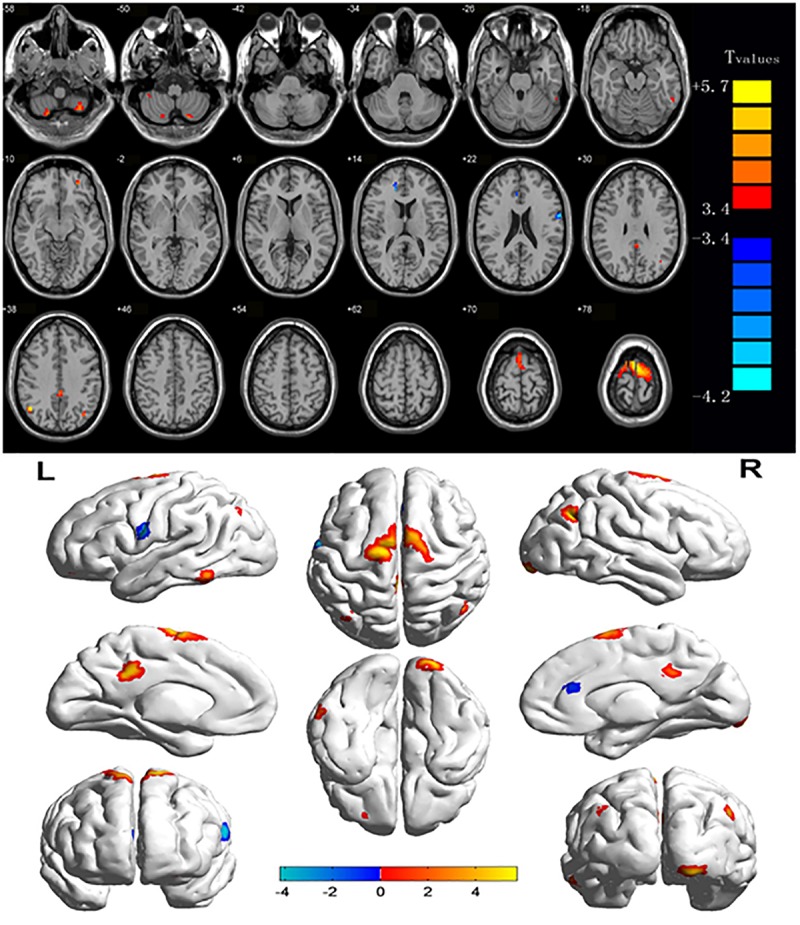
Significant differences in ReHo values between the CU group and HCs.

**FIGURE 2:**
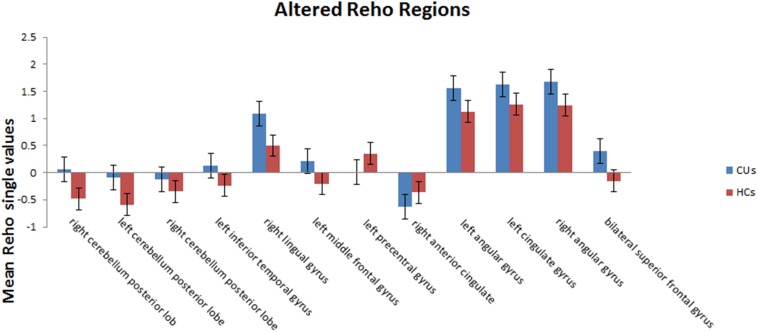
The mean single ReHo value between the CUs group and HCs. Data presented as mean ± standard deviation. ReHo, regional homogeneity; HCs, healthy controls; N/A, not applicable; CU, corneal ulcer.

Compared with HCs, CU patients had significantly increased ReHo values in right cerebellum middle frontal gyrus, left angular gyrus, left cingulate gyrus, right angular gyrus and bilateral superior frontal gyrus and decreased ReHo values in left precentral gyrus and right anterior cingulate (Figures 1, 2 and Table 2).

**TABLE 2:** Brain regions with significantly different ReHo values between the CUs and HCs.

**Condition**	**Left/right**	**Brain areas**	**BA voxels**	**MNI coordinates**			**Peak**	***T*-value**
				***X***	***Y***	***Z***		
**CUs > HCs**								
1	Right	Cerebellum posterior lobe	/	18	−69	−57	21	4.2291
2	Left	Cerebellum posterior lobe	/	−33	−63	−57	30	4.497
3	Right	Cerebellum posterior lobe	/	39	−45	−48	15	4.0563
4	Left	Left inferior temporal gyrus	20	−60	−48	−21	14	3.8959
5	Right	Lingual gyrus	18/17	24	−93	−15	31	4.9238
6	Left	Middle frontal gyrus	11	−27	48	−6	16	4.2676
7	Left	Angular gyrus	39	−39	−69	36	21	4.6547
8	Left	Cingulate gyrus	32	−3	−42	33	36	4.8645
9	Right	Angular gyrus	39	48	−63	39	22	5.742
10	/	Bilateral superior frontal gyrus	31	−3	−3	78	275	5.7209
**CUs < HCs**								
1	Left	Precentral gyrus	4	−57	−3	24	25	−4.1336
2	Right	Anterior cingulate	32	15	39	18	18	−4.2228

*Voxel-level: *P* < 0.01, GRF correction, cluster-level: *P* < 0.05. CU, corneal ulcer; HCs, healthy controls; BA, Brodmann area; MNI, Montreal Neurological Institute.*

### Receiver Operating Characteristic Curve

**FIGURE 3:**
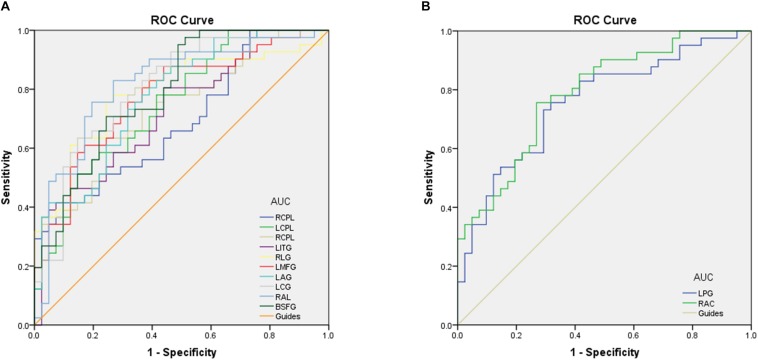
Receiver operating characteristic curve analysis of the ReHo values for altered brain regions. **(A)** The area under the ROC curve were RCPL 0.690 (*p* = 0.003; 95% CI: 0.577–0.803), LCPL 0.750 (*p* < 0.001; 95% CI: 0.646–0.853), RCPL 0.732 (*p* < 0.001; 95% CI: 0.624–0.839), LITG 0.726 (*p* < 0.001; 95% CI: 0.618–0.835), RLG 0.789 (*p* < 0.001; 95% CI: 0.689–0.890), LMFG 0.774 (*p* < 0.001; 95% CI: 0.673–0.875), LAG 0.776 (*p* < 0.001; 95% CI: 0.677–0.875), LCG 0.805 (*p* < 0.001; 95% CI: 0.710–0.901), RAL 0.817 (*p* < 0.001; 95% CI: 0.722–0.913), and BSFG 0.792 (*p* < 0.001; 95% CI: 0.697–0.888). **(B)** The area under the ROC curve were LPG 0.757 (*p* < 0.001; 95% CI: 0.652–0.861) and RAC 0.788 (*p* < 0.001; 95% CI: 0.691–0.884). ROC, receiver operating characteristic; ReHo, regional homogeneity; RCPL, right cerebellum posterior lobe; LCPL, left cerebellum posterior lobe; LITG, left inferior temporal gyrus; RLG, right lingual gyrus; LMFG, left middle frontal gyrus; LAG, left angular gyrus; LCG, left cingulate gyrus; RAL, right angular gyrus; BSFG, bilateral superior frontal gyrus; LPG, left precentral gyrus; RAC, right anterior cingulate.

To test whether the distinctive ReHo values detected from the CU and HC groups could differ CU patients from HCs, we performed the ROC curve analysis. The areas under the ROC curve (AUCs) for ReHo values were as follows: right cerebellum posterior lobe, 0.690; left cerebellum posterior lobe, 0.750; right cerebellum posterior lobe, 0.732; left inferior temporal gyrus, 0.726; right lingual gyrus,0.789; left middle frontal gyrus,0.774; left angular gyrus, 0.776; left cingulate gyrus, posterior lobe, left cerebellum posterior lobe, left inferior temporal gyrus, right lingual gyrus, left posterior lobe, left cerebellum posterior lobe, left inferior temporal gyrus, right lingual gyrus, left 0.805; right angular gyrus, 0.817 and bilateral superior frontal gyrus, 0.792 (CUs > HCs) (Figure 3A); left precentral gyrus, 0.757, and right anterior cingulate, 0.788 (CUs < HCs) (Figure 3B).

## Discussion

**FIGURE 4:**
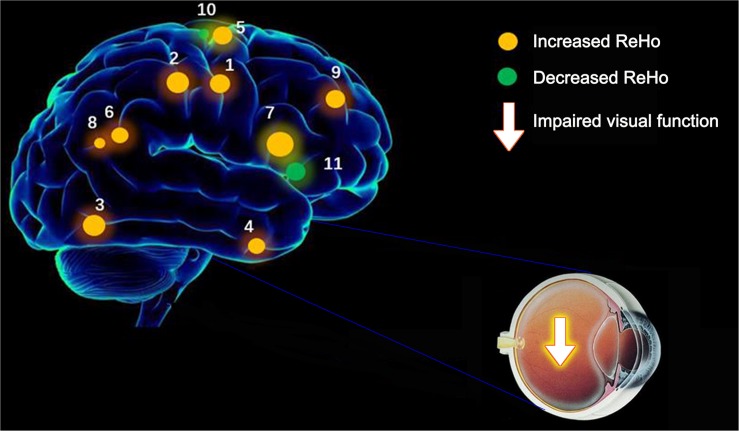
The ReHo results of brain activity in the CU group. Compared with the HCs, the ReHo of the following regions were decreased to various extents: 1-right cerebellum posterior lobe (*t* = 4.2291), 2-left cerebellum posterior lobe (*t* = 4.497), 3-left inferior temporal gyrus (*t* = 3.8959), 4-right lingual gyrus (*t* = 4,9238), 5-left middle frontal gyrus (*t* = 4.2676), 6-left angular gyrus (*t* = 4.6547), 7-left cingulate gyrus (*t* = 4.8645), 8-right angular gyrus (*t* = 5.742), 9-bilateral superior frontal gyrus (*t* = 5.7209), 10-left precentral gyrus (*t* = –4.1336), and 11-right anterior cingulate (*t* = –4.2228). The sizes of the spots denote the degree of quantitative changes. ReHo, regional homogeneity; HCs, healthy controls; CU, corneal ulcer.

This ReHo method has been successfully applied in several ophthalmological diseases and predicts huge development prospect (Table 3). As far as we know, this is the first study that the ReHo method has been used to estimate the effect of CU on resting-state brain activity till now. Compared to HCs, CU patients had significantly increased ReHo values in right cerebellum posterior lobe, left cerebellum posterior lobe, left inferior temporal gyrus, right lingual gyrus, left middle frontal gyrus, left angular gyrus, left cingulate gyrus, right angular gyrus and bilateral superior frontal gyrus, and decreased ReHo values in left precentral gyrus and right anterior cingulate (Figure 4).

**TABLE 3:** ReHo method applied in ophthalmological diseases.

**References**	**Disease**
Song et al., 2014	Glaucoma
Shao et al., 2015	Optic neuritis
Huang et al., 2016b	Strabismus
Cui et al., 2014	Diabetic retinopathy
Huang et al., 2016a	Open-globe injury
Huang et al., 2017b	Late monocular blindness
Huang et al., 2017a	Retinal detachment

### Analysis of the Increased ReHo Values in the CUs

Cerebellum posterior lobe (CPL), the portion of the cerebellum below the primary fissure, is the main motor control area. Previous research shows that CPL is associated with primary insomnia (Li P. et al., 2018), cognitive vulnerability to depression (Sun et al., 2018), and depressive disorder (Zhang Y. et al., 2018). In our study, the CU patients had increased ReHo values in left and right CPL, it may explain that sleeping dysfunction and depressive symptom sometimes occurred to the CU patients. Besides, Like CPL, many studies have shown that people with depression have dysfunction in the middle frontal gyrus (MFG) (Chang et al., 2011; Nelson et al., 2017). The MFG lies between the inferior frontal sulcus and the superior frontal sulcus, behind the precentral sulcus. This area is responsible for human cognitive and attention function (Martin et al., 2009). Our results showed that spontaneous neuronal activity was increased in MGF, it may also contribute to the depression in CU patients. Emotional disturbance in CU patients also includes dysphoria, schizophrenia and other unusual mental states, these disorders may relate to abnormal activities of lingual gyrus (LG), cingulate gyrus (CG), and superior frontal gyrus (SFG) in CU patients. LG is located between the sulcus of the callosum and the posterior part of the collateral sulcus. This region is believed to play a tremendously important role in vision and dreaming. Hypermetabolism in the region has been associated with visual snow syndrome and headache (Schankin et al., 2014). There were researches showed that LG activity changes dramatically in schizophrenia (Chen, 2018; Zhang P. et al., 2018). In our study, the CU patients had increased ReHo values in right lingual gyrus, which may explain the symptoms of headache and dysphoria happened in CU patients. CG is an important component of the limbic system. It can be divided into anterior cingulate cortex (AC) and posterior cingulate, two totally different areas of different functions. The former one is involved in many complex bodily, visceral motor functions and pain responses, and the later one monitors sensory and stereoscopic positioning and memory functions. Previous study has shown that the metabolism of CG can affect the emotional disorder (Shukla et al., 2018). In our study, the CU patients had increased ReHo values in left CG but decreased ReHo values in right AC, the mechanism still remains exploring. SFG, a part of the middle frontal gyrus. Associated with deliberative decision making, SFG has significantly and positively abnormal fMRI signals in individuals under acute social stress (Chang and Yu, 2018). It also has relationship with cognitive function (Zheng J. et al., 2018). In our study, the CU patients had increased ReHo values in bilateral SFC, it may help explain the unusual mental state of CU patient. Speaking of cognitive function, the CU patients had increased ReHo values in left inferior temporal gyrus (ITG) in our study. ITG, also known as gyrus temporalis inferior, lies in the convolution or protuberance of temporal lobe of the cerebral hemispheres, which is beneath the middle temporal sulcus and the ITG region stretches to the inferior sulcus. This area is known to be associated with behavioral learning and object memory based on current research. It is also responsible for visual perception, involved in facial recognition and believed to be associated with cognitive processes (Tu et al., 2018; Zheng W. et al., 2018). Therefore, apart from the cornea factor, the abnormal ITG and SFC activities may contribute to obstacle of item identification, too. Angular gyrus (AG) is an important associative region in the back of the brain above the Wernicke region and at the apex occipital lobe. It transfers visual information to Wernicke’s area, and understands the meanings of perceived words visually (Hall, 2010). It is also involves a number of processes associated with digital processing, language learning, spatial cognition, memory retrieval, and psychological theory. Previous research showed that attention-deficit was associated with abnormal AG activities (Kong et al., 2017). In our study, the CU patients had increased ReHo values in left and right AG, which may suggest that dysfunction of attention showed in CU is reasonable.

### Analysis of the Decreased ReHo Values in the CU

Precentral gyrus (PG), or anterior central gyrus, mostly lies on the lateral (convex) side of the cerebral hemispheres. Its borders are the precentral sulcus above; and the lateral fissure below. PG is the cortical motor area, and manages the opposite side half body to move at will. According to recent researches, this area also has relationship with depressive disorder (Li C. et al., 2018) and memory performance (Shang et al., 2018). In our study, the CU patients had decreased ReHo values in left PG, which may implicit the poor memory and black mood happened in CU. And the finding may explain the skin paresthesia of some CU patients.

Receiver operating characteristic curve proves the reliability of the results. The accuracy is perceived as perfect when AUC values is 0.7–0.9, a value between 0.5 and 0.7 is considered moderate, and less than 0.5 means the discrimination result is low. The ROC curve analysis in our study showed that the AUCs of each brain regions were over 0.7, which might represent these specific ReHo differences have a proper diagnostic accuracy in identifying CU. In brief, our findings demonstrated that the ReHo method might be a sensitive measurement of fMRI to diagnose CU patients in the future.

## Conclusion

In summary, the results showed that patients with CU had abnormal brain changes, performed as spontaneous activity. These findings provide important information for the understanding and lay a foundation of further study of the neural mechanisms underlying CU.

## Ethics Statement

The experiments were approved by the First Affiliated Hospital of Nanchang ethics committee. All the methods applied in the research followed the Declaration of Helsinki and complied with the medical ethics. For each subject, the research pact and process were fully explained, and written agreement was acquired.

## Author Contributions

YS contributed to the conception and design of the study. GT, C-QX, WW, and G-PG organized the database. TS, BL, X-WX, Y-LM, and W-fL carried out the statistical analysis. M-WX and H-ML wrote the first draft of the manuscript. All authors contributed to the manuscript revision, read, and approved its final version.

## Conflict of Interest Statement

The authors declare that the research was conducted in the absence of any commercial or financial relationships that could be construed as a potential conflict of interest.

## Footnotes

1 www.MRIcro.com

2 http://www.fil.ion.ucl.ac.uk/spm

3 http://rfmri.org/DPARSF

4 http://www.resting-fmri.Sourceforge.net
